# Supplementary material for: Hand and Oral Hygiene Practices among Adolescents in Dominican Republic, Suriname and Trinidad and Tobago: Prevalence, Health, Risk Behavior, Mental Health and Protective Factors
Source: Int J Environ Res Public Health. 2020 Oct 27;17(21):7860. doi: 10.3390/ijerph17217860 (PMC7663212; doi:10.3390/ijerph17217860)
Supplement: Supplementary file 1 [file ijerph-17-07860-s001.pdf]

Table S1. Description of study variables.

Variables	Question	Response Options (Coding Scheme)
Age	"How old are you?"	"11 years old or younger to 18 years old or older"
Sex	"What is your sex?"	"Male, Female"
Hunger	"During the past 30 days, how often did you go hungry because there was not enough food in your home?"	"1 = never to 5 = always (coded 1=0, 2-3=2 and 4–5=1)"
Oral hygiene	"During the past 30 days, how many times per day did you usually clean or brush your teeth?"	"1=I did not clean or brush my teeth during the past 30 days 6=4 or more times per day (coded 1-3=1 and 4-6=0)"
Hand hygiene	"During the past 30 days, how often did you wash your hands before eating?"	"1=never to 5=always (coded 1-4=1 and 5=0)"
	"During the past 30 days, how often did you wash your hands after using the toilet or latrine?"	"1=never to 5=always (coded 1-4=1 and 5=0)"
	"During the past 30 days, how often did you use soap when washing your hands?"	"1=never to 5=always (coded 1-4=1 and 5=0)"
	**Health risk behaviours**	
Current tobacco use	"During the past 30 days, on how many days did you smoke cigarettes/use any tobacco products other than cigarettes, such as such as country examples…?"	"1 = 0 days to 7 = All 30 days (coded 1=0 and 2-7=1)"
Trouble from alcohol use	"During your life, how many times have you got into trouble with your family or friends, missed school, or got into fights, as a result of drinking alcohol?"	"1=0 times to 4=10 or more times (coded 1=0 and 2-4=1)"
Current cannabis use	"During the past 30 days, how many times have you used marijuana?"	"1=0 times to 5=20 or more times (coded 1=0 and 2-5=1)"
Fruits	"During the past 30 days, how many times per day did you usually eat fruit such as …country examples?"	"1=I did not eat fruit during the past 30 days to 7=5 or more times per day"
Vegetables	"During the past 30 days, how many times per day did you usually eat vegetables, such as …country examples?"	"I did not eat vegetables during the past 30 days to 7=5 or more times per day "
Leisure-time sedentary behaviour	"How much time do you spend during a typical or usual day sitting and watching television, playing computer games, talking with friends, or doing other sitting activities, such as …country examples?"	"1=Less than 1 hour per day… 3= 3 to 4 hours per day …6=8 or more hours a day"
Physical activity	"Physical activity is any activity that increases your heart rate and makes you get out of breath some of the time. Physical activity can be done in sports, playing with friends, or walking to school. During the past 7 days, on how many days were you physically active for a total of at least 60 minutes per day?"	"0=0 days to 7=7 days (coded 0-6=0 and 7=1)"
No physical education	"During this school year, on how many days did you go to physical education (PE) class each week?"	"1=0 days to 6=5or more days (coded 1=1 and 2-6=0)"
	**Poor mental health**	

No close friends	"How many close friends do you have?"	"1 = 0 to 4 = 3 or more (coded 1+=0, 0=1)"
Lonely	"During the past 12 months, how often have you felt lonely?"	"1=never to 5=always (coded 1–3=0 and 4–5=1)"
Worry	"During the past 12 months, how often have you been so worried about something that you could not sleep at night?"	"1=never to 5=always (coded 1–3=0 and 4–5=1)"
Suicidal ideation	"During the past 12 months, did you ever seriously consider attempting suicide?"	"Yes, No"
Suicide attempt	"During the past 12 months, how many times did you actually attempt suicide?"	"1=0 times to 5=6 or more times (coded 1=0 and 2–5=1)"
	**Protective factors**	
School peer support	"During the past 30 days, how often were most of the students in your school kind and helpful?"	"1=never to 5=always (coded 1–2=1, 3=2 and 4–5=1)"
Parental supervision	"During the past 30 days, how often did your parents or guardians check to see if your homework was done?"	"1=never to 5=always (coded 1–3=0 and 4–5=1)"
Parental connectedness	"During the past 30 days, how often did your parents or guardians understand your problems and worries?"	"1=never to 5=always (coded 1–3=0 and 4–5=1)"
Parental bonding	"During the past 30 days, how often did your parents or guardians really now what you were doing with your free time?"	"1=never to 5=always (coded 1–3=0 and 4–5=1)"
Parental respect for privacy	"During the past 30 days, how often did your parents or guardians go through your things without your approval?"	"1=never to 5=always (coded 1–3=0 and 4–5=1)"
Non-parental tobacco use	"Which of your parents or guardians use any form of tobacco?"	"1=neither to 4=both (coded 1=1 and 2-4=0)"
